# Interaction of a pyrene derivative with cationic [60]fullerene in phospholipid membranes and its effects on photodynamic actions

**DOI:** 10.3762/bjoc.20.231

**Published:** 2024-10-30

**Authors:** Hayato Takagi, Çetin Çelik, Ryosuke Fukuda, Qi Guo, Tomohiro Higashino, Hiroshi Imahori, Yoko Yamakoshi, Tatsuya Murakami

**Affiliations:** 1 Department of Biotechnology and Pharmaceutical Engineering, Graduate School of Engineering, Toyama Prefectural University, 5180 Kurokawa, Imizu City, Toyama 939-0398, Japan; 2 Laboratorium für Anorganische Chemie, ETH Zürich, CH-8093 Zürich, Switzerland; 3 Department of Molecular Engineering, Graduate School of Engineering, Kyoto University, Nishikyo-ku, Kyoto 615-8510, Japan; 4 Instutite for Integrated Cell–Material Sciences (iCeMS), Kyoto University, Sakyo-ku, Kyoto 606-8501, Japan; 5 Instutite for Liberal Arts and Sciences (ILAS), Kyoto University, Sakyo-ku, Kyoto 606-8501, Japan

**Keywords:** liposome, π–π interaction, reactive oxygen species, superoxide radical anion

## Abstract

We have reported that upon visible light irradiation, ferrocene-porphyrin-[60]fullerene triad molecules yield long-lived charge-separated states, enabling the control of the plasma membrane potential (*V*_m_) in living cells. These previous studies indicated that the localization of the triad molecules in a specific intra-membrane orientation and the suppression of the photodynamic actions of the [60]fullerene (C_60_) moiety are likely important to achieve fast and safe control of *V*_m_, respectively. In this study, by mimicking our previous system of triad molecules and living cells, we report a simplified model system with a cationic C_60_ derivative (catC_60_) and a liposome with embedded 1-pyrenebutyric acid (PyBA) to demonstrate that the addition of PyBA was important to achieve fast and safer control of *V*_m_.

## Introduction

**Figure 1:**
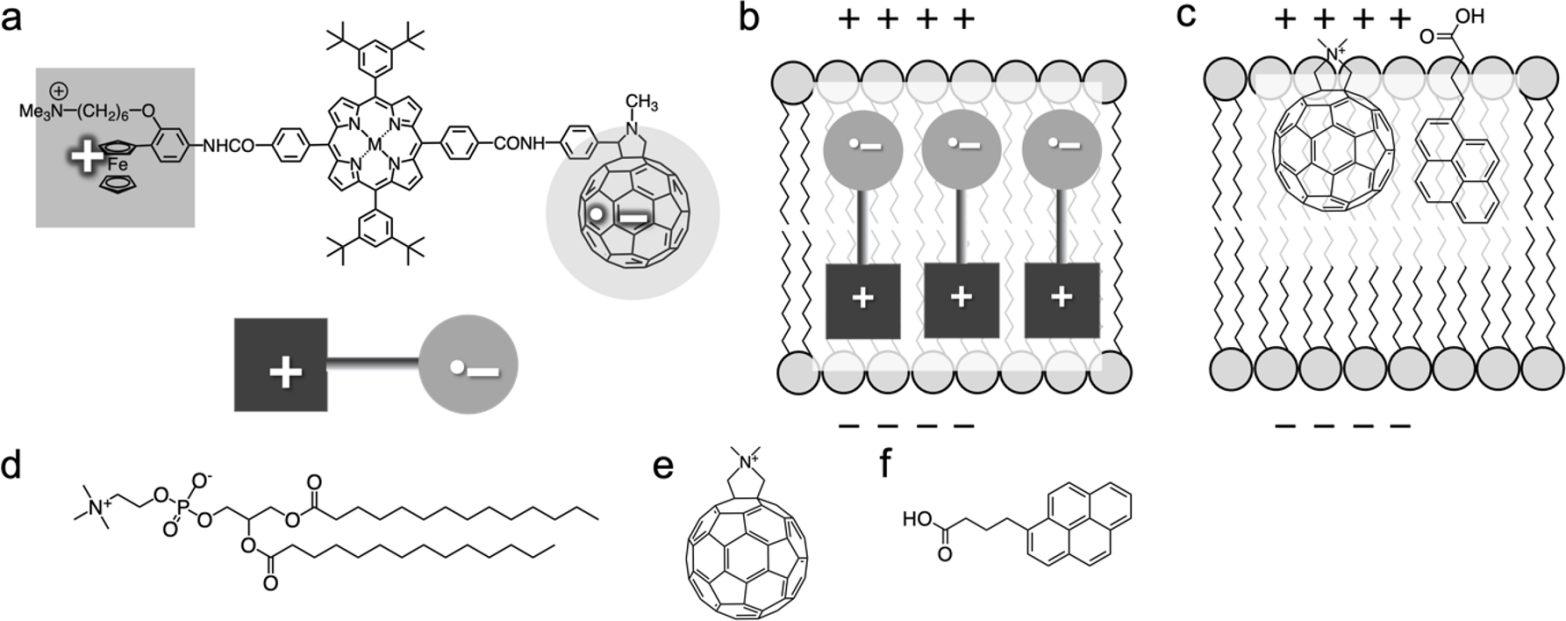
(a) Chemical structure and schematic illustration of the charge-separated state of a triad molecule and (b) control of the membrane potential by the illuminated molecule with a favourable arrangement. (c) Strategy of the present study for controlling both the location and photodynamic actions of a cationic derivative of C_60_ (catC_60_), a simple model compound of the triad molecules, in a membrane via π–π interactions with 1-pyrenebutyric acid (PyBA). (d–f) Chemical structures of 1,2-dimyristoyl-*sn*-glycero-3-phosphocholine (DMPC) (d), catC_60_ (e), and PyBA (f).

The [60]fullerene (C_60_) is known as an excellent electron acceptor [1–2] and is commonly used in organic solar cell applications [3]. Taking advantage of the fact that C_60_ can be an acceptor in photoinduced charge-separation systems, we have previously employed ferrocene-porphyrin-C_60_ triad molecules (Figure 1a) in a biological system to control the plasma membrane potential (*V*_m_) of living mammalian neuronal cells under photoirradiation [4–6]. Generally, *V*_m_ originates from a difference in electric charge on the two sides of the plasma membrane (approximately 5 nm thickness), with a slight excess of the positive ions inside relative to the negative ions outside. Our ferrocene-porphyrin-C_60_ triad molecule exhibited long-lived charge-separated states under visible light irradiation [7], with the C_60_ species becoming negatively charged while the ferrocene moiety became positively charged (Figure 1a). This charge-separated state can be used to initiate nanoscale electric fields, e.g., *V*_m_. The design of the triad molecules may also help to keep their orientation within the plasma membrane to have the C_60_ moiety located near the outer membrane surface and the ferrocene moiety near the inner membrane surface (Figure 1b). With this favorable arrangement of the molecules, it was expected to trigger a photoinduced change of the *V*_m_ that occurs at very fast time scales (less than milliseconds), leading to the (partial) cancellation of the *V*_m_. However, in reality, the change occurred on a minute time scale, indicating that the favorable arrangement was not sufficiently achieved in the plasma membrane.

One of the concerns with our previous triad molecules was photoinduced generation of reactive oxygen species (ROS) [8]. In our more recent study, the reversal of *V*_m_ after stopping photoirradiation of our triad molecule was associated with the renewal of the plasma membrane through endocytosis in living cells [6]. These results suggested that the photoinduced change in *V*_m_ was caused by some modification – most likely oxidation – of the plasma membrane by the photoexcited triad molecule. Taken together, for the realization of rapid control of *V*_m_ using such C_60_-based molecules in the membrane, the suppression of ROS generation is an important consideration. In this study, we aim to develop a system to achieve a quick *V*_m_ control without damaging the membranes by using a C_60_ derivative and a pyrene derivative as a model system for the triad molecules.

C_60_ has been reported to be incorporated into the phospholipid bilayers at the central part of membrane due to its hydrophobicity [9–10]. In contrast, to achieve the favorable arrangement as described above, the C_60_ moiety of the triad molecule needs to be located near the outer membrane surface. To facilitate this arrangement, in this study, we utilized a simplified system (Figure 1c) consisting of (i) liposomes of 1,2-dimyristoyl-*sn*-glyreco-3-phosphocholine (DMPC, Figure 1d), a well-known model of the plasma membrane, (ii) a cationic derivative of C_60_ (catC_60_, Figure 1e) as a replacement of the triad molecules, and (iii) 1-pyrenebutyric acid (PyBA, Figure 1f) as an anchor molecule for catC_60_ to be localized near the surface of phospholipid membranes [11–12]. With this model system, we aimed to examine whether both the intramembrane localization and the photodynamic actions of catC_60_ can be modulated by PyBA.

## Results and Discussion

**Figure 2:**
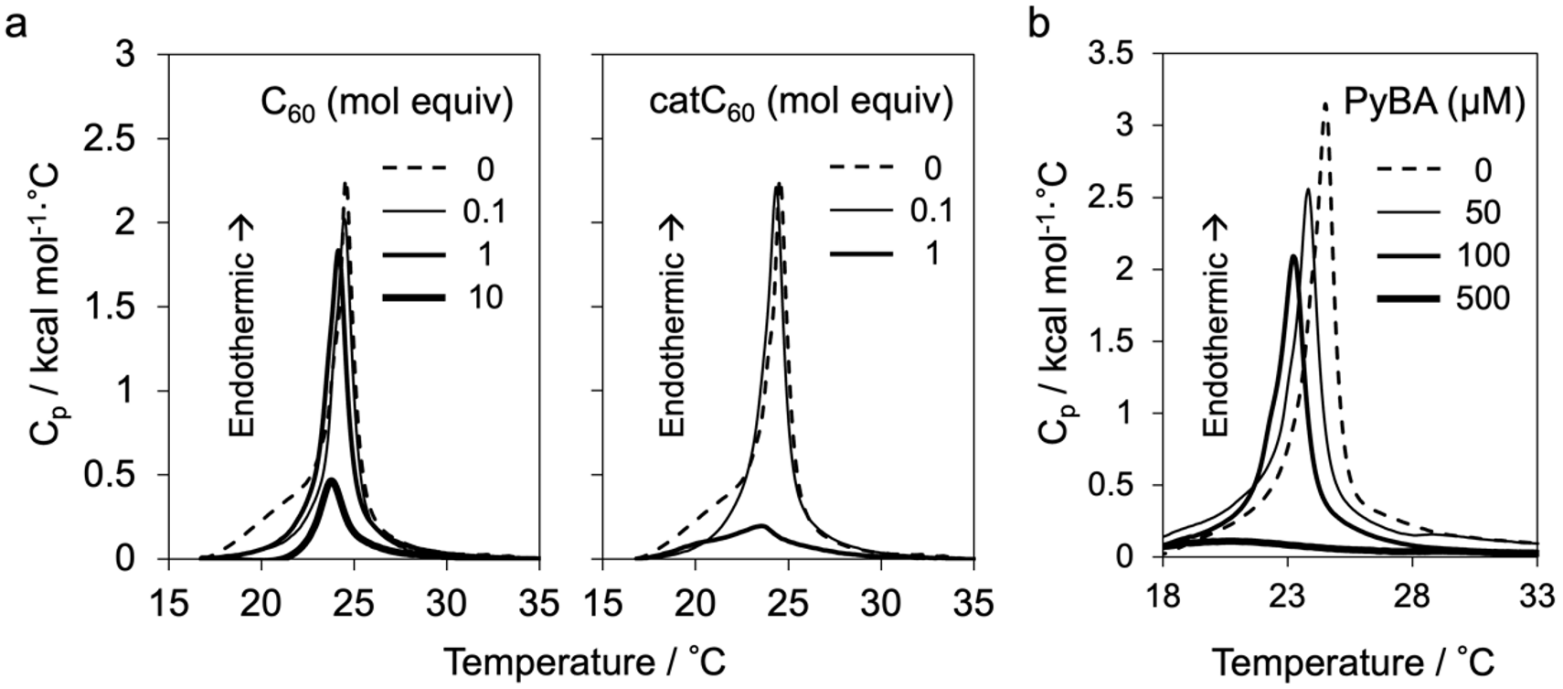
Differential scanning calorimetry analysis for the phase transition of liposomes (1 mM phospholipid). (a) Effect of the addition of C_60_ (left) or a cationic derivative of C_60_ (catC_60_) (right) at various molar equivalents (mol equiv) to the phospholipid of liposomes. (b) Effect of 1-pyrenebutyric acid (PyBA) addition at various concentrations to liposomes without catC_60_ and C_60_. The gel-to-liquid crystalline phase transition for 1,2-dimyristoyl-*sn*-glycero-3-phosphocholine (DMPC) liposomes was observed at 25 °C. All the measurements were performed with liposome samples dispersed in phosphate-buffered saline (PBS(–)).

The catC_60_-loaded liposomes (catC_60_-lip) were prepared by hydration of a catC_60_-embedded DMPC film [13] and compared with C_60_-loaded liposomes (C_60_-lip) by physicochemical characterizations. When catC_60_ or C_60_ was added to DMPC (in a 1:1 molar ratio to DMPC) the zeta potential of the catC_60_-lip was higher (16 mV) than that of C_60_-lip (–0.3 mV). Based on the experiments of differential scanning calorimetry analyses with varied amount of catC_60_ or C_60_ (Figure 2a), the addition of catC_60_ caused the disappearance of phase transition of DMPC liposomes in a dose-dependent manner and more efficiently than the case with C_60_. These results suggested that catC_60_ was more likely to localize near the surface of the lipid bilayer of catC_60_-lip than the C_60_ in C_60_-lip [14]. Similarly, the incorporation of PyBA into the pre-prepared liposomes was tested by zeta potential analysis (–15 mV) and differential scanning calorimetry analysis (Figure 2b), showing a clear dose dependency on the amount of PyBA added.

**Figure 3:**
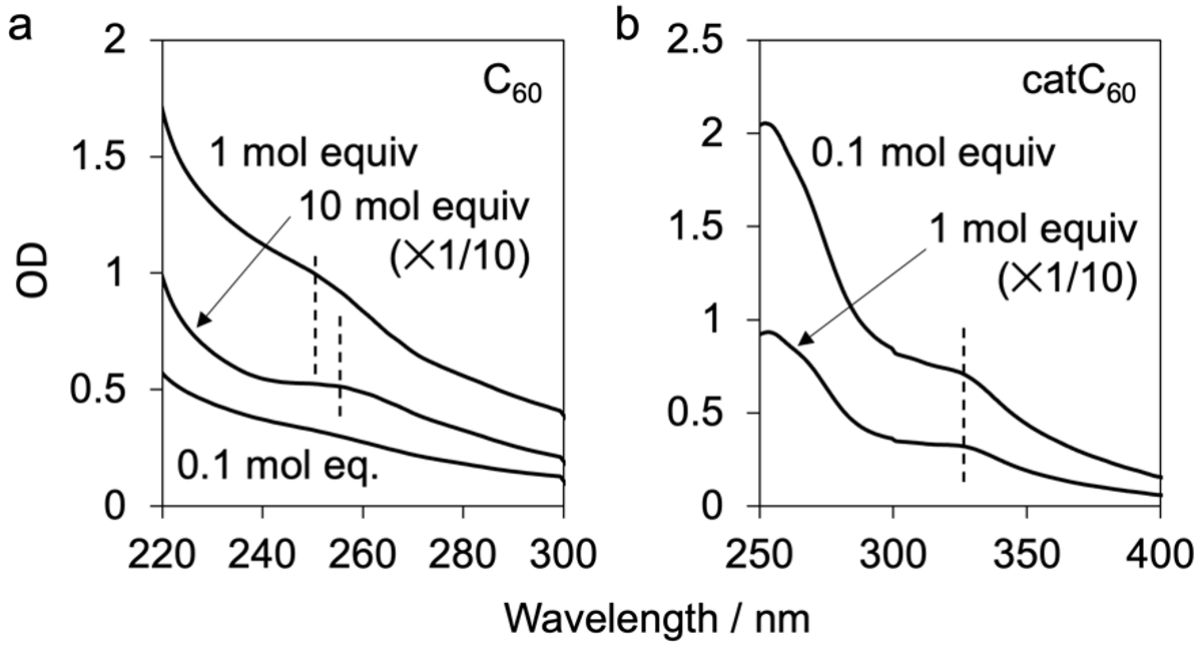
UV–vis absorption spectra of liposomes (1 mM phospholipid) with C_60_ (a) or a cationic derivative of C_60_ (catC_60_) (b) added at various molar equivalents (mol equiv) to phospholipid. The equivalent of C_60_ added in C_60_-loaded liposomes (C_60_-lip) was 0.1, 1 and 10 mol equiv, and the equivalent of catC_60_ added to catC_60_-loaded liposomes (catC_60_-lip) was 0.1 and 1 mol equiv. Spectra of 10 mol equiv of C_60_ in (a) and 1 mol equiv of catC_60_ in (b) were measured after 10-fold dilution. The absorption peak positions for C_60_ and catC_60_ are indicated with dotted lines. Liposome samples were dispersed in phosphate-buffered saline (PBS(–)).

The absorption spectra of catC_60_-lip and C_60_-lip were compared in PBS(–) (Figure 3). At two different concentrations, no significant change was observed in catC_60_-lip, whereas broadening and a red shift were observed in C_60_-lip at higher concentrations (10 mol equiv). These results indicate that catC_60_ was better dispersed in the DMPC membrane than C_60_. The results also provided some insight into the situation of our previous triad molecule – how the undesired aggregate formation of the triad molecules is reduced during solubilization and cell studies in physiologically relevant media [15].

**Figure 4:**
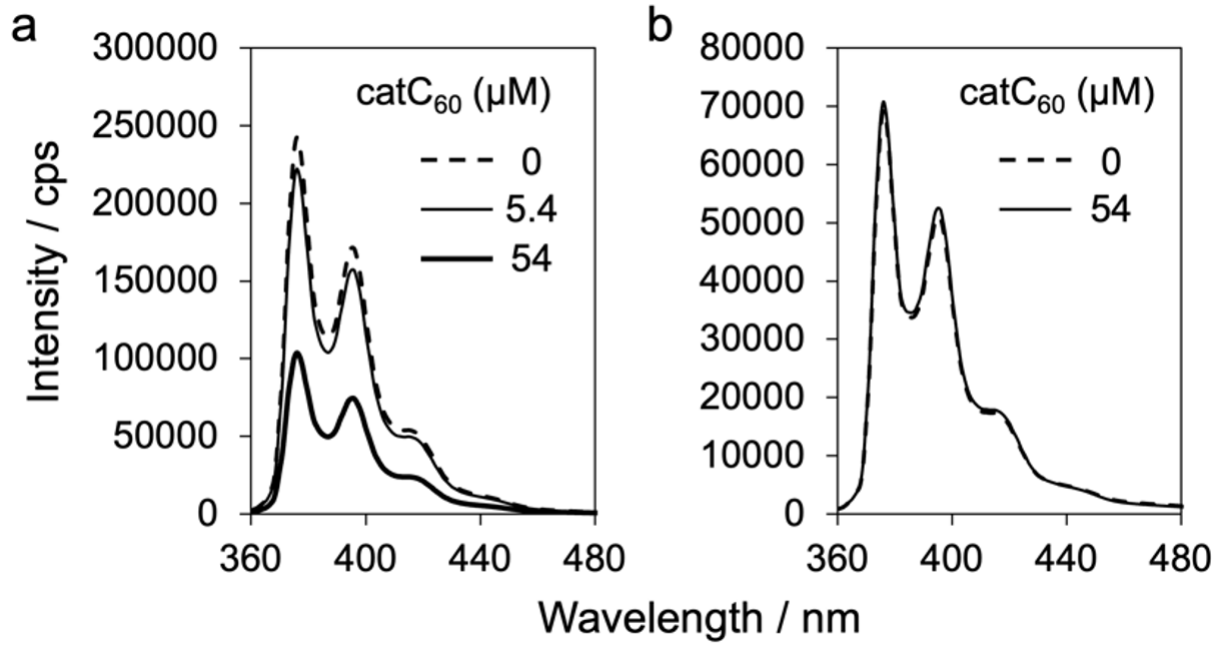
Fluorescence spectra of 1-pyrenebutyric acid (PyBA) in cationic derivative of C_60_ (catC_60_)-loaded liposomes (catC_60_-lip, 1 mM phospholipid) containing catC_60_ at various concentrations. (a) Effect of catC_60_ in liposomes on the fluorescence intensity of PyBA, with concentrations of catC_60_ at 0, 5.4, 54 µM, and PyBA at 50 µM. (b) Fluorescence spectra of catC_60_-lip with 0 and 54 µM catC_60_, treated with 50 µM PyBA, after addition of methanol. Liposome samples were dispersed in phosphate-buffered saline (PBS(–)).

The interaction between catC_60_ and PyBA in the liposomes was assessed by the fluorescence spectra of PyBA in catC_60_-lip [16]. The catC_60_-lip containing catC_60_ at 0, 5.4, and 54 µM were mixed with PyBA (50 µM) in PBS(–), and the fluorescence spectra were measured. As shown in Figure 4a, the intensity decreased upon increasing the concentration of the catC_60_ in the liposomes, showing the quench of PyBA fluorescence by catC_60_, presumably by interacting in the liposome membrane. The incomplete quenching after the addition of PyBA at a concentration comparable to that of catC_60_ may be attributed to the presence of unembedded PyBA in the dispersion. To this PyBA-embedded catC_60_-lip system, methanol was added to completely destroy the liposome structures, resulting in the regain of the fluorescence intensity (Figure 4b). The results clearly demonstrate that PyBA interacts with catC_60_ in the DMPC membrane near the surface, at least to some extent, indicating the potential of PyBA acting an anchor molecule to catC_60_ in the liposome membrane. Nevertheless, further study is necessary to gain more insight into their location in the membrane.

**Figure 5:**
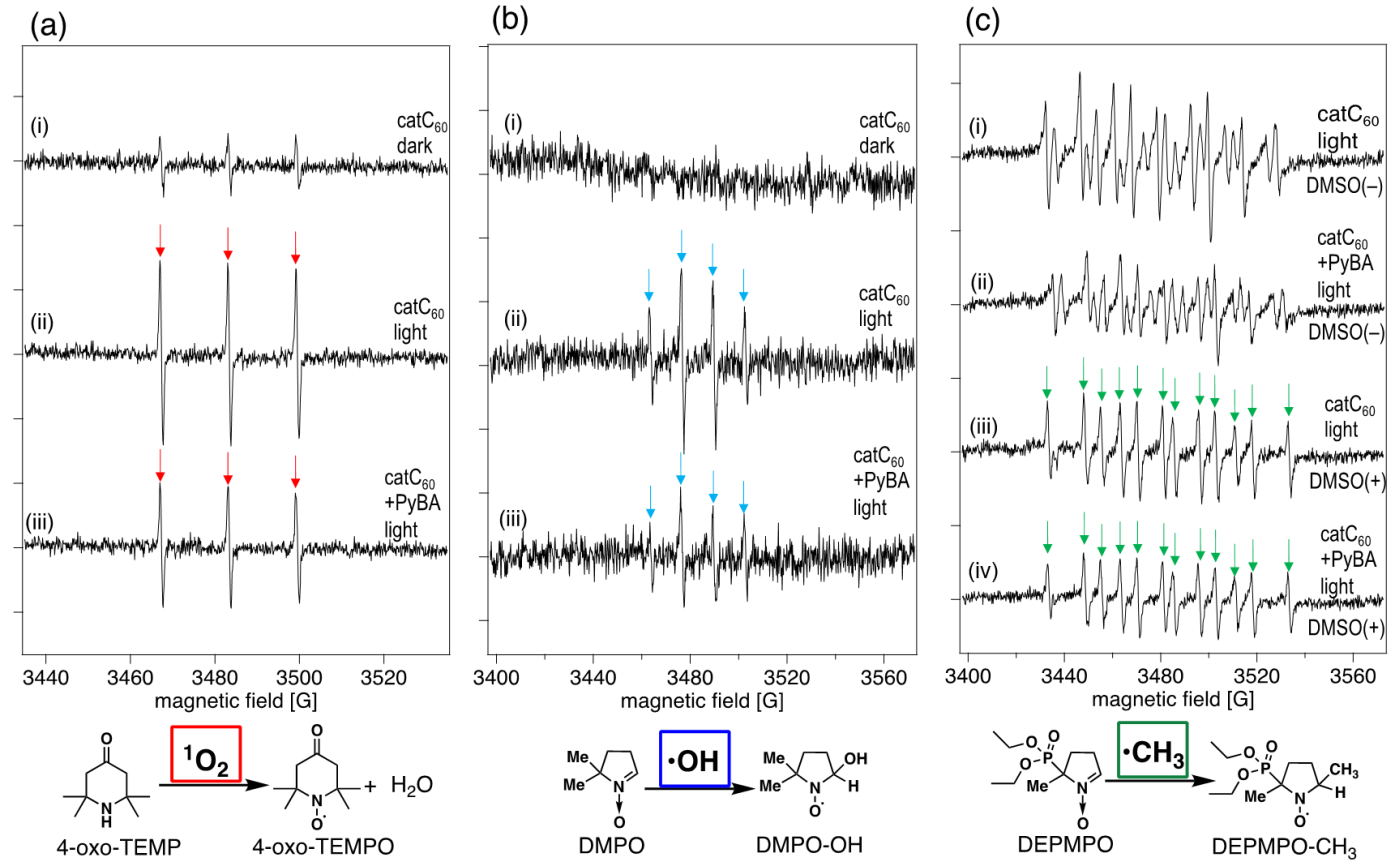
Photoinduced generation of reactive oxygen species (ROS) by cationic derivative of C_60_ (catC_60_)-loaded liposomes (catC_60_-lip) (5 µM catC_60_) in the absence and presence of 1-pyrenebutyric acid (PyBA, 50 µM). (a) X-band electron spin resonance (ESR) spectra of 2,2,6,6,-tetramethyl-4-piperidone (4-oxo-TEMP) adduct with ^1^O_2_ generated by catC_60_ under irradiation by a blue LED. Experimental conditions: (i) catC_60_ 5 µM and 4-oxo-TEMP 100 μM, in phosphate-buffered saline (PBS(–)) under dark conditions. (ii) catC_60_ 5 µM and 4-oxo-TEMP 100 μM in PBS(–) under irradiation for 30 min by blue LED lamp. (iii) catC_60_ 5 µM, PyBA 67 µM, and 4-oxo-TEMP 100 μM, in PBS(–) under irradiation for 30 min by blue LED lamp. (b) X-band ESR spectra of 3,4-dihydro-2,3-dimethyl-2*H*-pyrrole 1-oxide (DMPO) adduct with •OH generated by catC_60_ under irradiation by a blue LED. Experimental conditions: (i) catC_60_ 5 µM, β-nicotinamide adenine dinucleotide, reduced disodium salt hydrate (NADH) 8 mM, Fe(II)-diethylenetriaminepentaacetic acid (DETAPAC) 1 mM, and DMPO 100 mM in PBS(–) under irradiation for 30 min by blue LED lamp. (ii) catC_60_ 5 µM, NADH 8 mM, Fe(II)-DETAPAC 1 mM, and DMPO 100 mM in PBS(–) under irradiation for 30 min by blue LED lamp. (iii) catC_60_ 5 µM, PyBA 67 µM, NADH 8 mM, Fe(II)-DETAPAC 1 mM, and DMPO 100 mM in PBS(–) under irradiation for 30 min by blue LED lamp. (c) X-band ESR spectra of 5-(diethoxyphosphoryl)-5-methyl-1-pyrrolidone-*N*-oxide (DEPMPO) adduct with undefined radicals (i, ii) or •CH_3_ (iii, iv) generated by catC_60_ under irradiation by a blue LED. Experimental conditions: (i) catC_60_ 5 µM, NADH 8 mM, DETAPAC 1 mM, and DEPMPO 100 mM in PBS(–) under irradiation for 30 min by blue LED lamp. (ii) catC_60_ 5 µM, PyBA 67 µM, NADH 8 mM, DETAPAC 1 mM, and DEPMPO 100 mM in PBS(–) under irradiation for 30 min by blue LED lamp. (iii) catC_60_ 5 µM, NADH 8 mM, DETAPAC 1 mM, and DEPMPO 100 mM in a 4-to-1 (v/v) mixture of PBS(–) and dimethyl sulfoxide (DMSO) under irradiation for 30 min by blue LED lamp. (iv) catC_60_ 5 µM, PyBA 67 µM, NADH 8 mM, DETAPAC 1 mM, and DEPMPO 100 mM in a 4-to-1 (v/v) mixture of PBS(–) and DMSO under irradiation for 30 min by blue LED lamp. In the ESR spectra, signals corresponding to the adducts are indicated with red (4-oxo-TEMPO in a), blue (DMPO-OH in b), and green (DEPMPO-CH_3_ in c) arrows.

The results above indicated the interaction of catC_60_ with PyBA in the DMPC liposome membrane. We anticipated some effect of PyBA on the photoinduced generation of ROS by catC_60_ due to such interaction within the liposome membrane. To investigate such effects, we employed an electron spin resonance (ESR) spin-trapping method to evaluate the generation of ROS by catC_60_ in the absence or presence of PyBA. As spin trapping reagents for the singlet oxygen (^1^O_2_), hydroxyl radical (•OH) and superoxide radical anion (O_2_•^–^); 2,2,6,6,-tetramethyl-4-piperidone (4-oxo-TEMP), 3,4-dihydro-2,3-dimethyl-2*H*-pyrrole 1-oxide (DMPO), and 5-(diethoxyphosphoryl)-5-methyl-1-pyrrolidone-*N*-oxide (DEPMPO) were respectively used (schemes in Figure 5). Our previous study demonstrated that both ^1^O_2_ and O_2_•^–^ were generated under irradiation of triad molecules in DMSO/H_2_O [8].

Under irradiation by a blue LED (464–477 nm, 23 lm·W^–1^), significant ESR signals corresponding to the ^1^O_2_ adduct of 4-oxo-TEMP (4-oxo-TEMPO) were observed in the dispersion of catC_60_-lip ([catC_60_] = 5 µM) in PBS(–) showing an evidence of energy transfer reaction by the photoexcited catC_60_ (Figure 5a(ii)). In the presence of electron donor (NADH) under photoirradiation, •OH generation was observed as a •OH adduct of DMPO (DMPO-OH, Figure 5b(ii)) revealing that electron transfer reaction was also occurring. Using DEPMPO as a spin trapping reagent, detection of O_2_•^–^ was tried and some radical adducts were detected, but without being clearly identified (Figure 5c(i), (ii)). The reason of the inability of O_2_•^–^ detection is not known at present. Upon addition of dimethyl sulfoxide (DMSO) to this system, an adduct of DEPMPO and •CH_3_ (DEPMPO-CH_3_), which was presumably generated from the reaction of •OH and DMSO, was clearly observed, further confirming the generation of •OH (Figure 5b(iii)). At the same time, unusually fast conversion of O_2_^•–^ to •OH was also suggested in this system.

The results above suggest that catC_60_-lip generated both types of ROS (^1^O_2_ and •OH) via energy transfer and electron transfer mechanisms. The present results are in line with previous studies of photoinduced ROS generation by C_60_ and its derivatives [17–19]. The most important: upon the addition of PyBA to catC_60_-lip, the signal intensities of both types of ROS (^1^O_2_ and •OH) were decreased (Figure 5a(iii), b(iii), c(iv)). These results indicate that PyBA suppresses ROS generation by catC_60_-lip in liposome environment, which would be advantageous for the nanoscale control of *V*_m_ by the triad molecules.

## Conclusion

In summary, our findings indicate that PyBA can interact with catC_60_ within DMPC liposomes and modestly inhibit the photoinduced generation of ROS by catC_60_. These insights offer valuable guidance for the photocontrol of the plasma membrane potential (*V*_m_) using fullerene-containing triad molecules on a millisecond scale.

## Experimental

### Preparation of liposomes with catC_60_ (catC_60_-lip) or C_60_ (C_60_-lip)

Liposomes were prepared using a thin-film hydration method. DMPC (NOF AMERICA Corporation, White Plains, NY, USA) was solubilized in ethanol (FUJIFILM Wako Pure Chemical Corporation, Osaka, Japan), and catC_60_, which was synthesized according to a previous report [20], or C_60_ (NOF AMERICA Corporation, White Plains, NY, USA) was solubilized in a 1:4 (vol:vol) mixture of DMSO (Nacalai Tesque Inc., Kyoto, Japan) and toluene (FUJIFILM Wako Pure Chemical Corporation, Osaka, Japan). DMPC in ethanol and catC_60_ or C_60_ in DMSO/toluene were mixed in molar ratios of 1:0, 1:0.1, 1:1, or 1:10, and the solvent was removed using a rotary evaporator (Rotavapor R-300, BÜCHI Labortechnik AG, Switzerland) at 40 °C to prepare the lipid films. The lipid films were then dried overnight in vacuo. Then, the films were hydrated with PBS(–) (137 mM NaCl, 2.68 mM KCl, 8.1 mM Na_2_HPO_4_, 1.47 mM KH_2_PO_4_) so that the theoretical value of DMPC concentration was 3 mg/mL, and the resulting suspension was sonicated at 30 °C until the lipid membrane had completely peeled off from the flask. To remove free catC_60_ and C_60_, the resulting suspension was centrifuged at 20,000*g* at room temperature for 10 min*.* The supernatant was collected and subjected to more than 20 extrusions using a Mini-Extruder equipped with a 100 nm-pore-size membrane (Croda International Plc. Avanti Polar Lipids, Inc.).

### Differential scanning calorimetry (DSC)

DSC was performed using a MicroCal^TM^PEAQ-DSC System (Malvern Panalytical, Ltd., Malvern, U.K.). Liposomal suspensions of DMPC with or without catC_60_ or C_60_ were dispersed in PBS(–) (1 mM DMPC). Measurements were performed following equilibration at 10 °C at a scan rate of 180 °C/h. Measurements were also performed after mixing of 50, 100 or 500 µM PyBA (Sigma-Aldrich, St. Louis, MI, USA) and DMPC liposomes without catC_60_ or C_60_ followed by dialysis of the mixture against 3 L PBS for 2 h to remove free PyBA. Data analysis, including calculation of the phase transition temperature, was performed using the MicroCal PEAQ-DSC Software.

### UV–vis absorption measurement

UV–vis spectra of DMPC liposomes (1 mM DMPC) with or without catC_60_ or C_60_ were measured in PBS(–) using a UV-3600 Plus absorption spectrometer (Shimadzu Corporation, Kyoto, Japan).

### Fluorescence measurement

DMPC liposomes containing 0, 5.4, or 54 µM catC_60_ were mixed with PyBA (final concentration of 50 µM) in PBS(–), and the mixture was dialysed against 3 L PBS for 2 h to remove free PyBA. Fluorescence spectra were measured using an RF-6000 spectrofluorometer (Shimadzu Corporation, Kyoto, Japan) (excitation at 341 nm, emission at 360–500 nm) to evaluate the interaction between catC_60_ and PyBA in DMPC membranes. Measurements were also performed after the addition of 10 times the volume of methanol to the liposome samples to liberate catC_60_ and PyBA from the membranes.

### ESR measurements for photoinduced ^1^O_2_ and O_2_•^–^ generation

ESR spectra were recorded on a Bruker EMX, Continuous Wave X-Band EPR spectrometer (Bruker BioSpin GmbH, Rheinstetten, Germany). Suprasil^®^ ESR tubes with a diameter of 4 mm, length of 250 mm and a wall thickness of 0.8 mm were used (SP Wilmad-LabGlass, New Jersey, US). 4-Oxo-TEMP was purchased from ABCR (Karlsruhe, Germany) and purified by sublimation prior to use. The 50 µL Blaubrand^®^ intraMark capillaries were used in the EPR measurements (Brand GMBH, Wertheim, Germany). DEPMPO was bought from Enzo Life Sciences AG (Farmingdale, NY, USA). FeSO_4_, DETAPAC and NADH was bought from Sigma-Aldrich (St. Louis, Missouri, USA). DMPO was bought from TCI (Tokyo Chemical Industry Co. Ltd., Tokyo, Japan). Irradiation was performed by blue LED light (464–477 nm, 23 lm·W^–1^) from Lumiflex300 Pro RGB LED Stripes (LUMITRONIX LED-Technik GmbH, Hechingen, Germany) containing 120 LED lamps assembled in an aluminium cylindrical container with a diameter of 8.5 cm. ESR measurements conditions: microwave frequency 9.78 GHz, microwave power 10 mW, receiver gain 5.02 × 10^4^, modulation amplitude 1.00 G, modulation frequency 100 kHz, 3 scan average, sweep time 83.89 s.

**^1^O_2_ Generation:** All measurements were performed in PBS(–). Ten µL of catC_60_ sample solution (25 µM), 10 µL of 4-oxo TEMP solution (500 mM) and 30 µL of PBS(–) were mixed in a 0.5 mL Eppendorf tube. For the measurement in the presence of PyBA, 10 µL of PyBA solution (335 µM) was added instead of 10 µM of PBS(–). The solution was subjected to O_2_ bubbling for 30 seconds and then taken into 50 µL capillary and sealed. The solution was then irradiated with blue LED light for 30 minutes. The capillary was taken into the ESR tube for measurement at room temperature.

**^•^OH Generation:** All measurements were performed in PBS(–). Ten µL of catC_60_ sample solution (25 µM), 10 µL of Fe(II)-DETAPAC solution (5 mM), 10 µL of DMPO solution (500 mM), 10 µL of NADH (40 mM) and 10 µL PBS(–) were mixed in a 0.5 mL Eppendorf tube. For the measurement in the presence of PyBA, 10 µL of PyBA solution (335 µM) was added instead of 10 µL of PBS(–). The solution was subjected to O_2_ bubbling for 30 seconds and then taken into 50 µL capillary and sealed. The solution was irradiated with blue LED light for 30 minutes. The capillary was taken into the ESR tube and ESR spectra were recorded at room temperature.

**O_2_^•–^ Generation:** Measurements were performed in a mixture of DMSO and PBS(–) (1-to-4, v/v). Ten µL of catC_60_ sample solution (25 µM), 10 µL of DETAPAC solution (5 mM), 10 µL of DEPMPO solution (500 mM), 10 µL of NADH (40 mM) and 10 µL of PBS(–) were mixed in a 0.5 mL Eppendorf tube. For the measurement in the presence of PyBA, 10 µL of PyBA solution (335 µM) was added instead of 10 µL of PBS(–). The solution was subjected to O_2_ bubbling for 30 seconds and then taken into 50 µL capillary and sealed. The solution was irradiated with blue LED light for 30 minutes. The capillary was taken into the ESR tube and ESR spectra were recorded at room temperature.

## Data Availability

The data that supports the findings of this study is available from the corresponding author upon reasonable request.
